# Age-Related Macular Degeneration Is Associated with Less Physical Activity among US Adults: Cross-Sectional Study

**DOI:** 10.1371/journal.pone.0125394

**Published:** 2015-05-01

**Authors:** Paul D. Loprinzi, Bonnielin K. Swenor, Pradeep Y. Ramulu

**Affiliations:** 1 The University of Mississippi, Center for Health Behavior Research, Department of Health, Exercise Science, and Recreation Management, University, Mississippi, United States of America; 2 Wilmer Eye Institute, Johns Hopkins School of Medicine, Baltimore, Maryland, United States of America; Copenhagen University Hospital Roskilde and the University of Copenhagen, DENMARK

## Abstract

**Background:**

We have a limited understanding of the effects of age-related macular degeneration (AMD) on physical activity (PA), and we have no prevalence estimates of the daily movement patterns among Americans with AMD. Therefore, we examined the association between AMD and PA and provided estimates of the daily movement patterns of Americans with AMD.

**Methods:**

Data from the 2005-2006 National Health and Nutrition Examination Survey were used, including 1,656 adults (40-85 yrs). Retinal imaging was performed to classify individuals as no AMD, early AMD, or late AMD. Participants wore an ActiGraph 7164 accelerometer for 7 days to measure PA behavior.

**Results:**

93.2% of participants with late AMD were in the least desirable group (not sufficiently active and having a negative light intensity-sedentary behavior balance). After adjustments (including age), participants with late AMD, as compared to those with no AMD, engaged in 50% less moderate-to-vigorous physical activity (MVPA) (RR = 0.50; 95% CI: 0.28-0.90). When visual acuity was entered into the model along with the other covariates, the association between late AMD and MVPA was no longer significant (RR = 0.54; 95% CI: 0.29-1.01), suggesting that visual acuity may partially mediate this relationship.

**Conclusions:**

Individuals with late AMD engage in very little moderate-to-vigorous physical activity. Visually acuity, in part, explains the relationship between late AMD and PA.

## Introduction

Age-related macular degeneration (AMD) is the leading cause of vision loss among adults in industrialized countries [1,2], particularly older white Americans [3]. Early AMD occurs with milder stages of the “dry” form of the disease, marked by the presence of drusen and/or pigment epithelial changes. Late AMD occurs with the “wet” form of the disease (marked by choroidal neovascularization in the macula) or with advanced stages of the “dry” form of the disease, marked by geographic atrophy. In both stages of AMD vision is impaired, though to differing degrees. Vision loss associated with AMD may impair quality of life through various ways, including increased morbidity [4,5], difficulty recognizing faces, watching television, reading fine print [6,7], and decreasing functional independence [8,9].

Vision loss associated with AMD may also negatively influence other aspects of an individual’s health, as recent work demonstrates that those with vision impairment engage in less moderate-to-vigorous physical activity (MVPA) than those without vision impairment [10]. This is concerning, as insufficient participation in physical activity may increase the risk of developing metabolic, cardiovascular, and cerebrovascular diseases [11] that are associated with vision loss, systemic illness, and premature mortality [12,13].

Prior research has found that both AMD and impaired visual acuity are associated with reduced physical activity. One study has recently reported lower physical activity levels among those with impaired visual acuity [10]. Similarly, few studies have examined the association between AMD and physical activity. Results from these prior studies are mixed [14–19], with most studies demonstrating that AMD is associated with reduced physical activity levels. These prior studies have two major shortcomings: (a) none examine the association between AMD and physical activity after controlling for visual acuity; therefore, it is uncertain whether AMD or visual acuity is driving the potential relationship between AMD and physical activity; and (b) each of the 6 studies examining the association between AMD and physical activity have exclusively used self-report physical activity methodology. Previous work has shown that self-report physical activity is prone to considerable measurement error [20], and serious concerns have been raised regarding the validity of this method [21]. Validation studies examining the association between self-report physical activity and some gold-standard (e.g., accelerometry, indirect calorimetry, and doubly labeled water) typically show a poor correlation in the range of 0.3–0.5 [22]. Thus, these ‘validated’ self-report questionnaires only account for 9–25% of the variance in the outcome parameter and are therefore likely to result in considerable misclassification.

Here, we investigate the association between physical activity and AMD using an objective-measure of physical activity (accelerometry). Our search of the literature found no studies that have provided estimates of the daily movement patterns of Americans comparing those with and without AMD. Consequently, we have no information about the risk that these individuals may have for developing negative health outcomes associated with physical inactivity. Additionally, it is unknown if the association between AMD and physical activity is due to resulting impaired visual acuity.

This study hypothesizes that: a) the estimates of the daily movement patterns of American adults will be lower in those with AMD (particularly those with advanced AMD) as compared to without AMD; b) adults with AMD engage in less physical activity than those without AMD after accounting for potential confounders of this relationship; and c) this association will be partially explained by visual acuity impairment. To maximize the generalizability of our findings, we study these questions using a nationally representative sample of U.S. adults participating in the National Health and Nutrition Examination Survey (NHANES).

## Methods

### Design and Participants

NHANES is an ongoing survey conducted by the National Center for Health Statistics which evaluates a representative sample of non-institutionalized U.S. civilians. Participants are selected by a complex, multistage probability design. Data from the 2005–2006 NHANES were used, as this is the only NHANES cycle with both retinal imaging/grading and accelerometry data. All procedures for data collection were approved by the NCHS ethics review board, and all participants provided written informed consent prior to data collection. NHANES is publically accessible data and the authors used NHANES data for secondary analyses (http://www.cdc.gov/nchs/nhanes.htm). Participants were eligible for our analyses if they provided sufficient accelerometry data (≥ 4 days of 10+ hrs/day of monitoring data), had analyzable retinal imaging data, and provided data on the study covariates. In the 2005–2006 NHANES cycle, 2,413 participants were eligible for the retinal exam. After excluding those with insufficient accelerometry data or missing data on the covariates, 1,656 participants remained, which constituted the analytic sample. There were no differences in the study variables between the analytic sample and those excluded. Characteristics of study participants by AMD category are shown in Table 1.

**Table 1:** Weighted characteristics of the analyzed sample by age-related macular degeneration (AMD) severity, NHANES 2005–2006.

Variable	No AMD	Early AMD	Late AMD	P	η^2^
Age in years, mean (95% CI)	55.6 (54.1–57.0)	68.3 (66.1–70.6)	79.2 (76.1–82.3)	<0.001	0.93
Gender, % (95% CI)					
Male	48.1 (46.1–50.1)	50.4 (37.4–63.4)	31.2 (6.3–56.1)		
Female	51.8 (49.8–53.8)	49.5 (36.5–62.5)	68.7 (43.8–93.6)	0.42	0.05
Race-Ethnicity, % (95% CI)					
non-Hispanic white	79.1 (73.8–84.3)	85.6 (78.9–92.4)	100		
Other	20.8 (15.6–26.1)	14.3 (7.5–21.0)	0	0.09	0.15
BMI, mean kg/m^2^ (95% CI)	28.8 (28.2–29.4)	29.5 (28.3–30.7)	27.4 (25.0–29.8)	0.25	0.08
Mean arterial pressure, mean mmHg (95% CI)	90.0 (89.3–90.7)	90.1 (86.1–94.1)	93.3 (85.3–101.3)	0.82	0.05
Cotinine, mean ng/mL (95% CI)	58.5 (46.8–70.3)	34.3 (25.4–43.2)	15.4 (0.0–40.1)	0.005	0.41
Coronary heart disease, % (95% CI)					
No	95.1 (93.7–96.4)	89.7 (83.6–95.8)	93.8 (82.2–100.0)		
Yes	4.8 (3.5–6.2)	10.2 (4.1–16.3)	6.1 (0.0–17.7)	0.06	0.17
Stroke, % (95% CI)					
No	96.9 (96.2–97.7)	90.4 (83.4–97.4)	80.9 (59.9–100.0)		
Yes	3.0 (2.2–3.7)	9.5 (2.5–16.5)	19.0 (0.0–40.0)	0.003	0.31
Diabetes, % (95% CI)					
No	87.9 (85.2–90.6)	82.5 (72.6–92.3)	93.8 (82.2–100.0)		
Yes	12.0 (9.3–14.7)	17.4 (7.6–27.3)	6.1 (0.0–17.7)	0.21	0.09
Sedentary behavior (min/day)	494.7 (487.4–502.0)	527.8 (496.1–559.5)	573.7 (500.0–647.5)	0.04	0.25
Light-intensity physical activity (min/day)	348.1 (341.6–354.6)	317.8 (284.2–351.4)	252.6 (212.9–292.2)	<0.001	0.62
Moderate-to-vigorous physical activity (min/day)	21.4 (19.8–23.0)	14.2 (8.8–19.6)	3.9 (1.3–6.5)	<0.001	0.92
Daily movement patterns,% †				0.004	0.80
Group 1	12.2 (9.7–14.8)	10.5 (1.6–19.3)	0		
Group 2	27.0 (23.2–30.9)	10.5 (1.6–19.4)	0		
Group 3	9.0 (7.1–10.9)	5.7 (0.8–10.7)	6.7 (0.0–75.0)		
Group 4	51.5 (47.2–55.8)	73.1 (57.7–88.5)	93.2 (24.9–100.0)		
Accelerometer wear time, hr	14.4 (14.2–14.5)	14.3 (13.7–14.9)	13.8 (12.9–14.7)	0.20	0.10
Valid accelerometer days, %					
4 days	8.7 (7.2–10.4)	9.2 (1.8–16.5)	11.2 (0.0–27.7)		
5 days	17.9 (14.6–21.3)	10.0 (1.7–18.2)	3.7 (0.0–11.5)		
6 days	24.7 (22.0–27.4)	24.6 (16.6–32.6)	32.4 (5.6–59.2)		
7 days	48.4 (43.2–53.6)	56.1 (45.9–66.2)	52.6 (26.6–78.7)	0.24	0.08
Presenting better-eye visual acuity, mean logMAR (95% CI)	0.06 (0.05–0.07)	0.12 (0.09–0.15)	0.43 (0.28–0.57)	<0.001	0.64

† 4 daily movement patterns were assessed: Group 1) sufficiently active (see methods section) light-intensity physical activity (LPA) ≥ sedentary behavior (SED); Group 2) sufficiently active and but LPA < SED; Group 3) not sufficiently active but LPA ≥ SED; and Group 4) not sufficiently active and LPA < SED.

A Wald test was used to make comparisons between no AMD and late AMD across continuous variables (e.g., age). A chi-square test (design-based likelihood ratio test) was used for categorical variables (e.g., education). The design-based likelihood ratio test converts the chi-square value into an F-statistic. Beyond probability testing, the magnitude of each Wald test F-statistic and design-based likelihood ratio F-statistic was estimated using eta-squared (η ^2^), with values ≥ 0.1, 0.3, and 0.5, respectively, thought to be small, medium, and large (Lau & Kuk, 2011).Eta-squared (η^2^) calculated using the formula: (df_1_*F)/((df_1_*F)+df_2_). BMI = Body mass index. The 95% confidence intervals represent the 95% confidence interval for the observed mean/proportion. No AMD, n = 1533. Early AMD, n = 107. Late AMD, n = 16. AMD, age-related macular degeneration.

### Assessment of Age-Related Macular Degeneration

Participants aged 40 years and older were eligible for the retinal imaging exam unless they were unable to see light with both eyes open or had an eye infection. Detailed procedures of the retinal imaging exam performed in the NHANES 2005–2006 cycle can be found elsewhere (http://www.cdc.gov/nchs/nhanes/nhanes2005-2006/OPXRET_D.htm). Briefly, retinal imaging was performed using the Canon Non-Mydratic Retinal Camera CR6-45NM (Canon, Tokyo, Japan). Two forty-five degree digital images were obtained on both eyes and graded using the University of Wisconsin Age-Related Maculopathy Grading System for AMD. Further details can be found elsewhere (http://www.cdc.gov/nchs/data/nhanes/nhanes_05_06/NHANES_ophthamology_digital_grading_protocol.pdf). Using the worse eye in reference to AMD severity, early AMD was defined as either soft drusen greater than 500*μ* and a pigmentary abnormaility, or soft drusen within the center grading circle [diameter = 1 disc diameter; centered on the fovea] and a pigment abnormality. Late AMD was defined by the presence of any late lesions, such as choroidal neovascularization, subretinal fibrous scare, and/or geographic atrophy. Further details can be found elsewhere (http://www.cdc.gov/nchs/data/nhanes/nhanes_05_06/NHANES_ophthamology_digital_grading_protocol.pdf).

### Assessment of Physical Activity

Participants who were able to walk were asked to wear an ActiGraph 7164 accelerometer on their right hip for 7 days. As distributed by NHANES personnel, accelerometers were affixed to an elastic belt that was worn around the participant’s waist near the iliac crest. Accelerometry provides an objective measure of the intensity, frequency and duration physical activity. Participants were asked to wear the accelerometer during all activities except water-based activities and sleeping. The accelerometer measured the frequency, intensity, and duration of physical activity by generating an activity count proportional to the measured acceleration.

Time spent at different physical activity intensities was assessed over 1-minute intervals, with each minute classified as sedentary, light, moderate, or vigorous. Time spent in either moderate and vigorous activity was defined as moderate-to-vigorous physical activity (MVPA), as participants engaged in very little vigorous-intensity physical activity (mean = 0.71 min/day; SE = 0.07). Using the SAS code provided by the National Center for Health Statistics, an activity count below 100 counts per minute (i.e., 0 to 99) was used to classify sedentary behavior [23]; activity counts between 100 and 2019 counts per minute were used to classify time spent in light-intensity physical activity; activity counts between 2020 and 5998 counts per minute were used to classify time spent at moderate-intensity [24]; and activity counts at or greater than 5999 counts per minute were used to classify time spent at vigorous-intensity [24]. Only those participants with at least 4 days of 10 or more hours/day of accelerometer wear time were included in the analyses in order to make sure that data adequately captured habitual physical activity patterns [24]. To monitor the amount of time the device was worn, nonwear was defined by a period of a minimum of 60 consecutive minutes of zero activity counts, with the allowance of 1–2 minutes of activity counts between 0 and 100 [24]. Each participants average time spent per day in physical activity from valid accelerometry data were analyzed.

### Daily Movement Patterns

Using the accelerometry data, participants were defined as *sufficiently active* if they engaged in at least 150 minutes of moderate-intensity physical activity per week, 75 minutes of vigorous-intensity physical activity per week, or some combination of the two. Four mutually exclusive daily movement patterns were created to account for whether individuals were sufficiently active for weekly MVPA, and also to account for the importance of sedentary behavior and light-intensity physical activity (LPA) [25,26]: a) participants are sufficiently active and having a positive light-intensity physical activity-sedentary (LPA-SED) balance (i.e., LPA ≥ SED); b) participants are sufficiently active, but having a negative LPA-SED balance (i.e., LPA < SED); c) participants not sufficiently active, but having a positive LPA-SED balance; and d) participants not sufficiently active and having a negative LPA-SED balance. Conceptually, the four groups represent a continuum with participants in the first group considered to be the most active/desirable group and participants in the last group being considered the least active/desirable group. This novel daily movement pattern classification takes into consideration the emerging work demonstrating independent effects of sedentary behavior and light-intensity physical activity on health.

### Measurement of Covariates

Potential covariates were considered on the basis of previous research demonstrating an association with physical activity and AMD. Covariates considered included: age, gender, race/ethnicity, body mass index (BMI), mean arterial pressure (MAP), cotinine, coronary heart disease, stroke, diabetes, accelerometer wear time, number of valid accelerometer days and visual acuity.

Questionnaires were completed to assess age, gender, race/ethnicity, and comorbid illness. Comorbid illness (coronary heart disease, stroke, diabetes) were defined as present if patients reported that they had been told by a health care professional that they had the condition. Participants were also considered to have diabetes if they were taking insulin or diabetic pills to lower blood sugar, had a HgbA1_C_ of 6.5% or greater, or had a fasting glucose level of 126 mg/dL or higher. Body mass index was assessed from measured weight and height (kg/m^2^).

Blood pressure was measured up to 4 times, and the mean arterial pressure ([diastolic blood pressure x 2)+systolic blood pressure]/3) was calculated from the available blood pressure measurements. Serum cotinine was measured by an isotope dilution high performance liquid chromatography/atmospheric pressure chemical ionization tandem mass spectrometry.

Details regarding the NHANES vision assessment are described elsewhere [10]. Briefly, presenting visual acuity for each eye was assessed using the ARK-760 (Nidek Co Ltd, Tokyo, Japan), an autorefractor containing built-in visual acuity charts and participants wearing their presenting correction (if any). Visual acuity was also rechecked after auto-refraction in eyes with a presenting visual acuity worse than 20/25. Visual acuity of the better-seeing eye was used given that sight in the better eye is most relevant to disability in numerous visual disorders [27,28]; analyses were also computed with visual acuity as the worse-seeing eye, but results were similar, so visual acuity of the better-seeing eye was used. Presenting visual acuity of the better-eye was treated as a continuous variable expressed in logMAR units (logarithm of the minimum angle of resolution).

With regard to the classification of visual function, participants with presenting visual acuity of 20/40 or better in either eye were classified as having normal sight. Participants with presenting visual acuities worse than 20/40, but postrefraction visual acuity in either eye of 20/40 or better, were classified as having uncorrected refractive error (URE). Participants with visual acuities worse than 20/40 after autorefraction, or who self-reported not being able to see light with both eyes open, were classified as having vision impairment (VI). Participants with missing data for presenting acuity in both eyes, or with visual acuity worse than 20/40 in both eyes with no autorefraction in either eye, were excluded from the analysis as they were considered to have incomplete visual acuity data.

### Data Analysis

All statistical analyses were performed using procedures from sample survey data using Stata (version 12.0, College Station, TX) to account for the complex survey design used in NHANES. Mobile Exam Center (MEC) sample weights were used to provide nationally representative estimates. In an effort to maintain nationally representative estimates, the sample weights for those with 4 or more days of valid accelerometry data were ratio-adjusted to maintain the age, sex, and race-ethnicity distribution of the full sample.

The percentage of participants in each AMD category was determined for each of the four daily movement patterns described above. Negative binomial regression models were used to determine the association between AMD and MVPA, as MVPA time is an outcome variable expressed in integral minutes and was positively skewed. Coefficients from negative binomial models are expressed as rate ratios (RR), reflecting the relative rate of events (i.e. MVPA) associated with specific model elements over a specific period of time (i.e. day). Two models were computed, with Model 1 including all covariates with the exception of visual acuity, and Model 2 included these covariates plus visual acuity. This second model was used to determine whether visual acuity attenuated the association between AMD and MVPA. Multivariable linear regression models were used to determine the association between AMD with sedentary behavior and light-intensity physical activity, as sedentary behavior and light-intensity physical activity (outcome variables) were not severely skewed. Similar to the MVPA analyses, visual acuity was added to these models to determine if the association between AMD and sedentary behavior and light-intensity physical activity was attenuated. For all analyses, p < 0.05 was established as statistical significance.

## Results

Among the 1,656 participants, 183 had a presenting visual acuity worse than 20/40 (logMAR 0.3) and 15 had a presenting visual acuity of 20/200 or worse (logMAR 1.0) (not shown in tabular format). After autorefraction, and using the procedures reported elsewhere [29], 1,526, 99, and 31 participants, respectively, had normal vision, uncorrected refractive error, and vision impairment. In the sample with no AMD, 99.4% had normal vision or URE, with 0.6% having VI; in those with early AMD, 96.4% had normal vision or URE, with 3.6% having VI; among those with late AMD, 56.4% had normal vision or URE, with 43.6% having VI.

Participants with more severe AMD had worse presenting visual acuity (Table 1). In models also controlling for age, gender, race-ethnicity, BMI, cotinine, MAP, comorbid illness, accelerometer wear time and number of valid accelerometry days, participants with early (β = 0.03; p = 0.01) and late AMD (β = 0.31; p < 0.01) had worse visual acuity than those with no AMD.

As shown in Table 1, participants with no AMD, early AMD, and late AMD, respectively, spent an average of 494, 527, and 573 min/day in sedentary behavior. Participants in these groups also averaged 348, 317, and 252 min/day of light-intensity physical activity respectively. Lastly, MVPA estimates were 21, 14, and 4 min/day respectively. For all estimates, those with late AMD engaged in significantly (p < 0.05) more sedentary behavior and less physical activity (both light-intensity and MVPA) than those with no AMD, with large effect sizes (η^2^ ≥ 0.5) noted for light-intensity physical activity and MVPA. Very few participants with AMD, particularly those with advanced AMD, demonstrated more beneficial physical activity characteristics, as noted from the 4 daily movement pattern groups.

The association between MVPA and AMD was examined in multivariable negative binomial models (Table 2). Unadjusted results were as follows: participants with early and late AMD, respectively, engaged in 34% (RR = 0.66; 95% CI: 0.46–0.95) and 72% (RR = 0.18: 95% CI: 0.09–0.34) less MVPA as compared to those with no AMD. After controlling for age, gender, race-ethnicity, BMI, cotinine, MAP, comorbid illness, accelerometer wear time an number of valid accelerometer days (model 1), participants with late AMD, as compared to those with no AMD, engaged in 50% less MVPA (RR = 0.50; 95% CI: 0.28–0.90). When visual acuity was entered into the model (model 2), the association between MVPA and late AMD was no longer statistically significant, though the magnitude and direction of the association was similar (RR = 0.54; 95% CI: 0.29–1.01), suggesting that visual acuity may be accounting for 8% ([0.50–0.54/0.50]*100) of the total effect between AMD and MVPA. AMD was not significantly associated with sedentary behavior or light-intensity physical activity in the multivariate models (data not shown).

**Table 2:** Weighted regression analyses examining the association between moderate-to-vigorous physical activity (outcome variable) and AMD, NHANES 2005–2006.

	Rate Ratio (95% CI)	
Variables	Model 1 †	Model 2 ‡
Age-related macular degeneration		
Early AMD vs. None	1.09 (0.75–1.58)	1.09 (0.75–1.58)
Late AMD vs. None	**0.50 (0.28–0.90)**	0.54 (0.29–1.01)
Covariates		
Age, per year increase	**0.95 (0.95–0.96)**	**0.95 (0.95–0.96)**
Female vs. Male	**0.60 (0.53–0.67)**	**0.60 (0.53–0.67)**
Other vs. non-Hispanic white	0.90 (0.76–1.07)	0.90 (0.76–1.07)
BMI, 1 kg/m^2^ higher	**0.95 (0.94–0.96)**	**0.95 (0.94–0.96)**
Cotinine, 1 ng/mL higher	**0.99 (0.99–0.99)**	**0.99 (0.99–0.99)**
Mean arterial pressure, 1 mmHg higher	1.00 (0.99–1.01)	1.00 (0.99–1.01)
Comorbid Illness		
Coronary Heart Disease vs. No Coronary Heart Disease	0.95 (0.70–1.29)	0.95 (0.70–1.29)
Stroke vs. No Stroke	0.81 (0.54–1.22)	0.81 (0.54–1.22)
Diabetes vs. No Diabetes	0.84 (0.70–1.02)	0.84 (0.70–1.02)
Accelerometer wear time, hr	**1.06 (1.02–1.10)**	**1.06 (1.02–1.10)**
Valid accelerometer days, #		
5 vs. 4 days	0.97 (0.71–1.33)	0.97 (0.71–1.33)
6 vs. 4 days	0.94 (0.75–1.16)	0.94 (0.75–1.16)
7 vs. 4 days	1.06 (0.85–1.29)	1.06 (0.85–1.29)
Presenting better-eye visual acuity, per 0.1 logMAR increase	N/A	0.98 (0.93–1.03)

† Included all covariates except for visual acuity.

‡ Same as Model 1 except visual acuity added to the model.

Bold indicates statistical significance (p < 0.05). AMD = age-related macular degeneration. BMI = Body mass index.

## Discussion

The aims of this study were to a) descriptively classify the daily movement patterns among U.S. adults with and without AMD and b) examine the association of AMD with physical activity while controlling for visual acuity along with other confounding variables. In a nationally representative sample of U.S. adults, individuals with late AMD spent significantly less time engaging in MVPA than those with early AMD or without AMD. Further, the majority of participants with early or late AMD engaged in the least desirable movement patterns, i.e., were not sufficiently active and engaged in more sedentary behavior than light-intensity physical activity. After including visual acuity to our analytic models, we found that the association between late AMD and MVPA was attenuated and no longer significant. We calculated that visual acuity may be accounting for 8% of the total effect of late AMD on physical activity.

The hypothesized role of visual acuity on the association between AMD and MVPA was based on the association between AMD and impaired visual acuity, as well as prior research indicating that vision loss may reduce physical activity participation. With regard to the latter, and although findings are mixed [14–19], most studies demonstrate an association between AMD and self-reported participation in physical activity, though no study has examined the association between AMD and objectively-measured physical activity. This potential reduction in MVPA may occur through various mechanisms, including decreased balance and fear of falling [30], which ultimately may affect their ability and/or inclination to engage in MVPA. Further, impaired balance associated with reduced visual acuity may specifically be due to worse vestibular balance, postural instability and problems with edge detection and navigation [30]. This is concerning, as insufficient participation in physical activity may increase the risk of developing various cardiovascular disease risk factors and other chronic diseases. Not only does physical inactivity increase the occurrence of various deleterious metabolic and vascular diseases, but, in theory, physical inactivity may be considered prodegenerative and facilitate the progression of vision loss and AMD in some cases [19], i.e., from sedentary behavior-induced increases in inflammation and endothelial dysfunction [31,32], which is supported by recent research [33]. Consequently, it is plausible to suggest a bidirectional relationship between physical activity and AMD [19].

While this is the first study to examine the role of visual acuity on the association between AMD and physical activity, there are potential limitations that should be considered. Although accelerometry is considered the standard of choice for measuring free-living physical activity behavior, accelerometry has notable limitations including an underestimation of physical activity from certain modes of physical activity, such as cycling or swimming. However, this limitation may be less of a concern among adults as their most common type of physical activity is walking [34]. Further, it is possible that other unmeasured factors may be influencing the association between AMD and physical activity which could affect the observed level of attenuation attributed to visual acuity. But, there is no statistical test to definitively determine the presence of unmeasured confounders that may influence this association. We can only make speculations as to what potential unmeasured factors were not collected in NHANES. One such factor may be the duration of vision loss, as this may influence emotional distress. Individuals having a shorter period of perceived vision loss are more likely to report higher levels of emotional distress [35], and among those with AMD, emotional distress influences quality of life and the ability to carry out daily activities [35]. Individuals with AMD often experience significant emotional distress, even when the severity of vision loss is considered [36]. Additionally, these are cross-sectional analyses and we are unable to assess the temporality of the association between visual acuity declines and physical activity. Further longitudinal studies, particularly those with a greater number of individuals with late AMD (only 16 participants in the present study had late AMD), are needed to address both the potential influence of duration of vision loss and to clarify the directionality of the association between physical activity and advanced AMD.

A strength of the study is the use of a nationally representative study population, which makes the results generalizable to the U.S. population. Furthermore, objective measures of visual acuity and physical activity were employed unlike previous studies which employed self-reported physical activity. Finally, disease diagnoses were formally made using retinal photographs and a centralized reading center.

In conclusion, the present findings demonstrate that adults with late AMD engage in less MVPA than those without AMD, and this association may be partially explained by decreased visual acuity. Additionally, regardless of the direction of causation, adults with AMD are more likely to engage in unhealthy daily lifestyle movement patterns, underscoring the importance of promoting physical activity and reducing sedentary behavior among adults with AMD. Although speculative, our null findings between AMD and sedentary/light-intensity physical activity behavior suggest that those with AMD do not appear to be restricting their light-intensity physical activity behavior or engaging in more accumulated sedentary behavior. This suggests that a more feasible approach to promoting movement-based behaviors among those with AMD would be to, at least initially, focus on increasing their number of breaks from sedentary behavior (e.g., minimizing prolonged sedentary behavior by, every hour, standing and walking for a few minutes), which, encouragingly, has been shown to beneficially associate with several health outcomes [37]. The inactive lifestyle among this population is particularly concerning as inactivity is associated with increased risk of cognitive decline in adults with AMD [38]. Encouragingly, promotion of physical activity among those with AMD may be feasible given that individuals with AMD do not appear to have worse mobility function than normal sighted individuals of the same age [39]; however, careful reflection and promotion of physical activity among those with late AMD, in particular, is needed as those with late AMD have worse vision and engage in less physical activity than their counterparts. Future longitudinal research further assessing the role of visual acuity decline on the association between AMD and physical activity, as well as examining the effect of the duration of vision loss, is warranted.

## Supporting Information

S1 STROBE Checklist (PDF)
